# Visualizing cognitive processes in medical education: Forward and backward reasoning in a digital family medicine assessment

**DOI:** 10.3205/zma001859

**Published:** 2026-06-15

**Authors:** Johanna Klutmann, Constanze Dietzsch, Ute Schlasius-Ratter, Alexander Oksche, Sara Volz-Willems, Sandra Jordan, Johannes Jäger, Fabian Dupont

**Affiliations:** 1 Saarland University, Department of Family Medicine, Homburg (Saar), Germany; 2 German Institute for State Examinations in Medicine, Pharmacy, Dentistry and Psychotherapy (IMPP), Mainz, Germany

**Keywords:** clinical reasoning, family medicine, forward reasoning, backward reasoning, undergraduate medical education

## Abstract

**Background:**

When evaluating medical students, wouldn’t it be helpful to visualize and understand your students’ clinical reasoning (CR)? There are different types of CR for students to use in their multiple-choice questions (MCQs). While forward reasoning uses data to generate a hypothesis, backward reasoning looks at possible prompts (answers) to generate a hypothesis. This study implements a new approach to visualizing CR during digital MCQ assessment. Furthermore, it examines the effect of feedback given during the learning process, also known as formative feedback, on the thought process.

**Methods:**

Quantitative and qualitative data were collected at the end of two consecutive year 5 end-of-semester Family Medicine exams in 2023. Both exams consisted of 60 MCQs and an additional research component also comprising MCQs. During the research component students digitally recorded their reasoning process while answering MCQs. Qualitative data were coded via three rounds of coding, including two marking/coding parties.

**Results:**

This study was able to digitally visualize CR in a large cohort (n=210). On average, the exam questions were answered with 87% of CR. Forward reasoning was used significantly more often than backward reasoning (WS 22/23 p=0.006, SS 23 p<0.001). High performers were significantly more likely to use forward reasoning and backward reasoning than low performers (p<0.01). Formative Feedback had no significant influence on the choice of CR type (p=0.281). Follow-up questions might stimulate a change in CR behaviour; however further research is needed (p<0.001).

**Conclusion:**

This study illustrates an alternative method to visualize students’ cognitive processes on a large scale. This approach sheds light on the required cognitive processes. It may help educators to better understand what to focus on during curricular learning activities aimed at preparing for state exams. This method may be beneficial as a quality criterion for MCQ questions, as it does not only rely on expert opinion or question metrics but illustrates students’ cognitive processes when answering MCQ.

## Zusammenfassung

**Hintergrund:**

Wäre es bei der Bewertung von Medizinstudierenden nicht hilfreich, das klinische Denken (CR) Ihrer Studierenden zu visualisieren und zu verstehen? Es gibt verschiedene Arten von CR, die Studierende in ihren Multiple-Choice-Fragen (MCQs) verwenden können. Während beim Vorwärtsdenken Daten zur Erstellung einer Hypothese verwendet werden, werden beim Rückwärtsdenken mögliche Hinweise (Antworten) zur Erstellung einer Hypothese herangezogen. Diese Studie implementiert einen neuen Ansatz zur Visualisierung von CR während der digitalen MCQ-Prüfung. Darüber hinaus untersucht sie die Auswirkungen von Feedback während des Lernprozesses, auch bekannt als formatives Feedback, auf den Denkprozess.

**Methoden:**

Quantitative und qualitative Daten von zwei Semesterabschlussprüfungen im Fach Allgemeinmedizin wurden am Ende des 5. Studienjahres im Jahr 2023 gesammelt. Beide Prüfungen bestanden aus 60 MCQs und einem zusätzlichen Forschungsteil, welcher ebenfalls MCQs umfasste. Während des Forschungsteils erfassten die Studierenden ihren Denkprozess bei der Beantwortung der MCQs digital. Die qualitativen Daten wurden in drei Kodierungsrunden, darunter zwei Markierungs-/Kodierungspartys, kodiert.

**Ergebnisse:**

Diese Studie konnte CR in einer großen Kohorte (n=210) digital visualisieren. Im Durchschnitt wurden die Prüfungsfragen mit 87% CR beantwortet. Vorwärtsdenken wurde signifikant häufiger verwendet als Rückwärtsdenken (WS 22/23 p=0,006, SS 23 p<0,001). Leistungsstarke Studierende verwendeten signifikant häufiger Vorwärtsdenken und Rückwärtsdenken als leistungsschwache Studierende (p<0,01). Formatives Feedback hatte keinen signifikanten Einfluss auf die Wahl der CR-Art (p=0,281). Folgefragen könnten eine Veränderung des CR-Verhaltens bewirken; jedoch sind weitere Untersuchungen erforderlich (p<0,001).

**Schlussfolgerung:**

Diese Studie veranschaulicht eine alternative Methode zur Visualisierung der kognitiven Prozesse von Studierenden in großem Maßstab. Dieser Ansatz beleuchtet die erforderlichen kognitiven Prozesse. Er kann Pädagogen dabei helfen, besser zu verstehen, worauf sie sich bei curricularen Lernaktivitäten zur Vorbereitung auf staatliche Prüfungen konzentrieren sollten. Diese Methode kann als Qualitätskriterium für MCQ-Fragen von Vorteil sein, da sie sich nicht nur auf Expertenmeinungen oder Fragenmetriken stützt, sondern auch die kognitiven Prozesse der Studierenden bei der Beantwortung von MCQs veranschaulicht.

## Introduction

Clinical reasoning (CR) is a core competency in medical education representing the thought process behind diagnosing and treating patients [1], [2], [3], [4], [5]. However, visualizing or assessing CR, especially in undergraduate medical education, remains a challenge [6].

Although CR is central to medical practice and education, traditional assessment methods - especially MCQs, which dominate high-stakes exams, are criticized for not properly capturing the cognitive processes involved in CR [7], [8]. Despite innovations like Key-feature Questions (KFQs), little is known about how CR can actually be mapped or visualized within standard MCQ assessments [9].

At the same time, many national licensing exams wish to maintain MCQs for their objectivity, standardization, and cost efficiency [10], [11]. Given the widespread use and high stakes MCQ assessment it’s crucial to understand and visualize how these questions stimulate or reflect CR during those assessment periods [12], [13]. Mapping CR during these assessments may significantly enhance both educational quality and clinical preparedness for students transitioning into the clinical field.

Previous research has distinguished between forward reasoning (FR) and backward reasoning (BR) [14] with FR being often described as the hallmark of expertise and deeper understanding [15]. FR describes the thought process in which students answer questions without having to read through the choices, generating their hypothesis from the MCQ question stem and possible added material [3]. BR describes thinking backwards and drawing on the answer choices (distractors) to answer the question instead [3]. The distinction between FR and BR only captures one aspect of how CR can be described. It focuses on how the reasoning process was formed. However, CR is a multidimensional construct. It can also be understood in terms of its goals, performance and contextual factors, all of which can be the focus of analysis [2], [4]. 

Approaches such as KFQs and Formative Feedback (FF) have been introduced to promote CR during assessments [9]. KFQs focus on one difficult aspect of solving a problem, often embedding this feature in a written case followed by a limited number of questions [16]. FF is a core element of “assessment for learning” [17]. It provides feedback during the learning process with a focus on assisting learning [17]. FF deepens students’ understanding, even during assessments [18].

To date, educators use MCQ metrics to describe MCQ items and their quality. These metrics offer valuable insights into how items perform on a population level – identifying which questions are too easy, too hard, or particularly effective at distinguishing between high- and low-performing students [19]. However, these measures are independent of students’ cognitive thought processes [20]. They provide no information about how or why a student arrived at a particular answer. Exploring observable reasoning processes rather than MCQ metrics could provide new insights into how CR is elicited, assessed, and supported during MCQ-based exams.

Other studies have called for a better understanding of CR utilization during assessment [21], [22]. This study investigates whether CR can be visualized during an undergraduate MCQ exam and how it relates to performance. Additionally, the influence of FF and follow up key feature questions (KFQ) on the usage of CR are analysed.

The aim of this study is to examine whether CR processes can be identified in the context of an undergraduate MCQ examination and to explore their relationship to student performance. More specifically, we analyse whether the use of CR, in particular FR and BR, is associated with higher performance across different item types and if and to what extent FF and follow-up key feature questions (KFQ) elicit different reasoning strategies.

## Methods

### Setting and study participants

In this mixed methods study, all participants were year five undergraduate medical students at Saarland University (UdS). The exam was the end of year compulsory Family Medicine exam, a digital state exam based MCQ assessment. It contained two identical exam setups including 60 MCQs, at the end of the winter semester 2022/2023 (WS 22/23) and at the end of the summer semester 2023 (SS 23). Both exams contained a research component, which consisted of two KFQs. These KFQs were case studies with follow-up questions. The KFQs used were not the same. Firstly, to include a wider range of questions in the study. Secondly, to prevent students from already knowing the questions due to possible student-internal discussions about the questions. In this study, the questions will be referred to by an abbreviation. The first number indicates the case study number and the second indicates the follow-up question number. After each follow-up question of the KFQ, there was an open-ended text box question, asking for a self-assessment of whether the students derived the question clinically and their cognitive thought process used in the previous question. The same structure applied to the SS23 exam. Additionally, in SS23, students received FF for the first time during an exam for a selection of questions. The FF was uniform information-based feedback consisting of the correct answer and a brief explanation. The participants were randomly divided into two groups: A (n=63) and B (n=52). Group A received FF after each follow-up question of the second KFQ and Group B received FF after each follow-up question of the first KFQ. In both groups, FF was provided in reverse order, to maintain face validity and comparability of CR (with and without FF). Ethical clearance was provided before the study (234/20-14.04.2022). The participants consented to the use of their study and exam performance before the exam.

### MCQ item selection 

In cooperation with the Institute for State Examinations in Medicine, Pharmacy, Dentistry and Psychotherapy (IMPP), a panel of two research students (JK, CD) and four Family Medicine faculty (SJ, SVW, FD, JJ) selected the follow-up KFQs from an MCQ pool provided by the IMPP based on the learning objectives of the semester. The follow-up KFQs used were divided into procedural, diagnostic, and factual questions.

### Data collection and analysis

**Figure 1:**
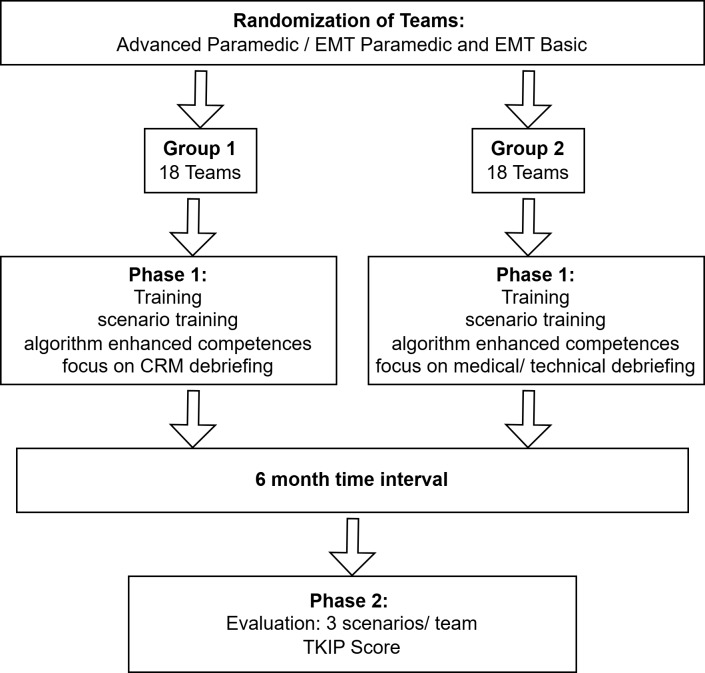
Qualitative deductive content analysis with three levels of coding

Both quantitative and qualitative data were collected and then exported to Excel (version 16.96.1). Qualitative data from both exams were analysed using three levels of deductive content analysis (see figure 1 (Fig. 1)), informed by a structured literature review. First, coders applied Young et al.'s framework to determine the presence of CR [2]. The aforementioned study identified six categories of terms regarding CR on which there was a consensus. These were: the purpose/goal of reasoning; the outcome of reasoning; reasoning performance; reasoning processes; reasoning skills; and the context of reasoning [2]. These categories and their associated themes served as a guideline to determine CR in our study. Second, they assessed the correctness and coherence of reasoning, focusing on clarity and conclusiveness rather than accuracy alone. Correct CR did not only mention an action, but the action is justified in the context of clinical logic. Whereas in an incorrect CR response essential clinical considerations were missing or overlooked and therefore a wrong conclusion was made. Third, correct CRs were categorized as FR or BR following Beullens et al. [3]. This study gave an example to distinguish FR and BR: An example of a data-driven reasoning [BR] statement is: “If he has an elevated blood sugar, then he must have diabetes”. An example of a hypothesis-driven reasoning [FR] statement is: “Because he has diabetes, he has an elevated blood sugar“ [3]. The coding guidelines also included the aspect to analyse if students justify their answers solely based on the answers provided (BR), or if the answer resulted from a more extensive clinical thought process (FR). The application of preexisting factual knowledge was coded as FR as a fast form of CR. This was based on the aspect of the Croskerry model, that repeated system 2 analyses, can become automatic system 1 responses (e.g., pattern recognition) [23]. Initially, coders (JK, CD, USR, SH, FD, JJ) were trained via video and discussion. All had prior qualitative coding experience. Each response was coded independently by all six coders. In a second session, codes with at least 4/6 agreement were accepted; discrepancies were discussed until full consensus was reached.

Quantitative data analysis was performed with jamovi^TM^ version 2.4.12. The analysis included descriptive statistics of FR and BR frequencies, a median split to define performance groups, and (χ^2^) tests to examine group differences and CR types regarding follow-up questions and FF. Binomial/multinomial logistic regressions tested whether follow-up questions or FF predicted CR type. 

## Results

The following results describe patterns of CR usage and differences in performance across two cohorts of students taking exams. It also examines the effects of feedback on one of these cohorts. In WS 22/23 95 out of 97 participated, in SS 23 115 out of 115 participated. The average performance of the exam was comparable for the two semesters, with average scores of 80% (WS 22/23) and 84% (SS 23) respectively. In total 4.95% of the answers in WS 22/23 and 1.44% of the answers in SS23 were skipped.

### The use of clinical reasoning 

CR was observed across both exams. An example of an answer coded as CR regarding the diagnosis of an obstructive lung disease, was: “I looked at the Tiffeneau Index. Furthermore, inhalation of salbutamol did not result in any reversibility of the obstruction. This led me to the diagnosis of obstructive ventilation disorder.” (WS.21.40). An exemplary response coded as No CR regarding the same question was “[I] cannot clearly identify either obstruction or restriction; therefore [I] simply took a mixture.” (WS.21.73).

**Figure 2:**
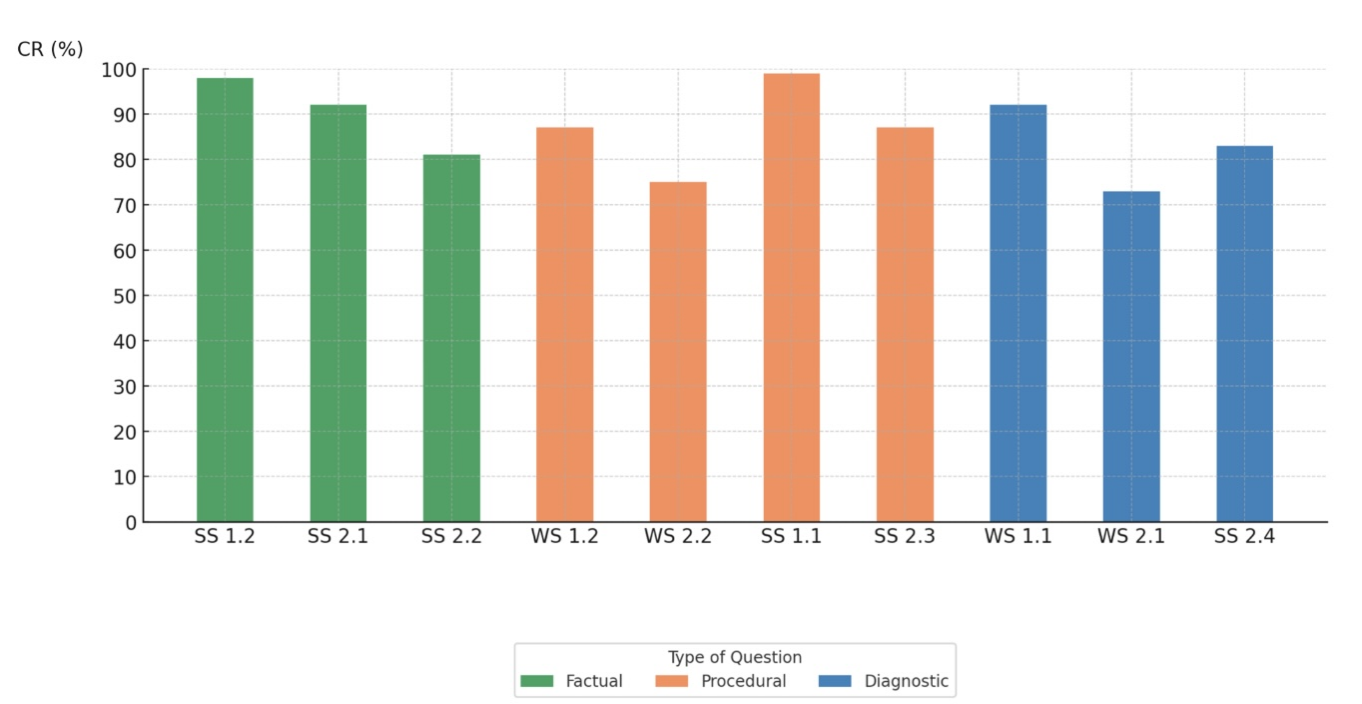
CR usage by question type and exam. Exam questions are categorized by semester (WS or SS) and number of the KFQ case study and follow-up question

On average, the exam questions were answered with 87% CR. As can be seen in figure 2 (Fig. 2), the highest proportion of CR usage was found in a procedural question (99.1%), while the lowest was noted in a diagnostic question (73.5%). In all questions, CR was applied more frequently than no CR. 

**Figure 3:**
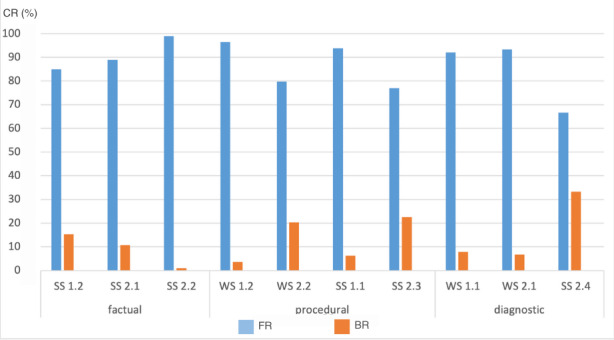
FR (Forward Reasoning) and BR (Backward Reasoning) usage compared between the three question types (factual, procedural, diagnostic) in summer- and winter semester (WS or SS) and number of the KFQ case study and follow-up question

The highest proportion of FR usage was found in a factual question (98.9%), while the lowest was noted in a diagnostic question (66.7%) (see figure 3 (Fig. 3)). For BR, the highest usage was in a diagnostic question (33.3%) and the lowest in a factual question (1.1%). FR occurred significantly more often than BR, both in WS 22/23 (p=0.006) and SS 23 (p<0.001). Two examples of coding for FR and BR regarding the further course of action in acute appendicitis were for FR: “Appendicitis should be treated surgically as quickly as possible; therefore, fasting upon admission to hospital” (SS.11.39) and for BR: “None of the other possible answers would have been appropriate in the case of acute appendicitis” (SS.11.96).

The fast form of FR, which consisted of the application of preexisting knowledge was coded in 3.5% of the FR answers. 

### High performers were more likely to apply CR

Students scoring at or above the median were classified as high performers and those below as low performers. High performers were more likely to apply CR (FR and BR) than low performers. The odds of using FR and BR were 3.3 (OR=3.33, 95% CI [2.378, 4.490], p<0.001) and 2.7 times higher (OR=2.67, 95% CI [1.669, 4.261], p<0.001), respectively, for high performers. In one question in WS 22/23, the high performers exclusively used FR. Moreover, students who applied any type of CR were more likely to answer a question correctly compared to students who did not use CR (p<0.001). 

### Feedback did not influence the use of CR

A binomial logistic regression showed no significant correlation between FF and the use of CR (χ^2^=1.78, p=0.182). CR occurred neither more frequently after FF nor after no FF. A multinomial logistic regression showed no significant influence of FF on the choice of CR type (χ^2^=3.38, p=0.281). Neither the comparison between BR and “no CR” (p=0.099), nor the comparison between FR and “no CR” (p=0.227) reached statistical significance.

### Higher probability of BR in follow-up KFQs in one semester 

In SS 23, a significant difference was found in the frequency of FR and BR between the first questions (1.1, 2.1) and the follow-up KFQ (1.2, 2.2, 2.3, 2.4) (χ^2^=256, p< 0.001). Students applied BR significantly more often for follow-up questions than for the first question. The probability of BR was about 2.5 times higher when it was a follow-up question compared to a first question (OR=2.49, 95% CI [2.29, 2.84], p<0.001). In WS 22/23, there was no significant difference in the frequency of FR and BR in relation to the first or follow-up questions (χ^2^=1.08, p=0.299). 

## Discussion

This study yielded four main findings. First, family medicine students were able to demonstrate CR within a tablet-based assessment format. Second, high-performing students were more likely to apply CR, and the use of CR was associated with a better performance. Third, FF had not significantly influenced the type of CR employed. Fourth, follow-up questions were associated with an increased likelihood of BR.

The results demonstrate that CR can be effectively captured and analysed in a digital assessment environment. CR processes were visualized across an entire academic year, enabling simultaneous data collection from a large cohort during the examination. This approach offers a valuable alternative to traditional post-exam interviews and may reveal more efficiently students’ CR processes. Contrary to common assumptions, the data suggests that medical students do not simply focus on answer options in MCQs [24]. Instead, many actively engage in reasoning processes, as FR was used significantly more frequently than BR. In a factual question, FR was used most often. This may be attributed to the fact that a large proportion of students remember factual answers from learnt information. The application of preexisting factual knowledge was coded as FR as a fast form of CR. This is comparable to pattern recognition of the Croskerry model [23]. Fewer students used FR in more complex questions. Consistent with existing literature, high performers relied more on FR than low performers [3]. This supports the notion that FR is more typical for expert reasoning patterns [15]. This could mean that the use of FR is situation dependent and that its application may increase with advancing competency levels (experts). These findings reinforce the importance of CR not only in clinical practice but also in undergraduate assessments. When students employed CR to answer a question, the likelihood of a correct answer increased. There is also evidence that FR may be more common among students who are able to think flexibly and apply knowledge creatively. For example, in WS 22/23, in one question high performers exclusively used FR. According to the panel that selected the questions, this item required “thinking outside the box” and went beyond standard diagnostic or therapeutic content, promoting students to draw on clinical imagination or real-world experience such as internships.

However, modern question types such as KFQs, which include follow-up questions, do not inherently promote FR. On the contrary, follow-up questions can lead students to prematurely focus on a single hypothesis and the preset answer options. This phenomenon, known as premature closure, limits the consideration of differential diagnoses and leads to search satisfaction – where reasoning halts after identifying one plausible diagnosis [25]. This may explain the increased likelihood of BR in response to follow-up questions in SS23. On the other hand, in WS 22/23, there was no significant difference in the frequency of FR and BR between the first and follow-up questions. This revealed a difference between the two cohorts, illustrating how the structure and type of assessment items can influence the reasoning process. The use of different questions, coupled with an imbalanced mix of factual, diagnostic and procedural questions highlights the importance of carefully considering item design in medical examinations. Seemingly minor variations in question format can substantially influence whether students engage in FR or BR strategies. Even though both semesters were subject to the same family medicine curriculum, it is important to consider the possibility that the reasoning strategies employed in the two semesters may have been influenced by differing practical experiences. In addition, disparities in learning motivation or stress management may underpin the observed variation in the frequency of BR. In situations of uncertainty, the propensity to avoid errors may be augmented, thereby facilitating a process of thinking backwards or the promotion of BR. Group psychology that develops within a cohort through exchange, mentors, or so-called "exam myths" could also influence the reasoning strategies of the cohorts. While innovative competency-based formats may aim to assess higher-order reasoning, they do not automatically elicit authentic CR. 

Similarly, the use of FF did not enhance CR in this study. While FF is known to support learning [17], [18], the design of the FF may influence its effectiveness. In the assessment, students received uniform information-based feedback consisting of the correct answer and a brief explanation. Research shows that CR skills improve when feedback includes specific suggestions for improvement, regardless of students’ performance [26]. The lack of individualization or absence of suggestions for improvement may explain why the FF did not significantly influence the usage of CR or FR.

To confirm these findings, further research with larger and more diverse cohorts at different stages of undergraduate medical education is needed. Future studies should explore the impact of KFQ formats and varied feedback types across disciplines, while continuing to visualize CR at scale. 

### Limitations 

The study focused on year five medical students within a Family Medicine curriculum in Germany. Family medicine, being a broad and integrative subject, may have contributed to the high point average (average scores of 80% and 84%). This could explain the high use of CR in general and FR in particular. Consequently, the data of this study may not be generalizable to students in other years, disciplines, or institutions. Furthermore, reasoning types (FR and BR) were coded based on students’ written explanation of their reasoning, focusing on their initial train of thought. Although six independent coders were involved and inter-rater agreement of at least two-thirds was achieved for all responses, it is possible that nuances of reasoning were lost in the process. There is also potential for desirability bias. Another limitation of this study is that factual questions were only present in one of the two cohorts (SS23). The examination questions were selected from an IMPP item pool according to curricular relevance, while the categorization into factual, diagnostic, and procedural domains was only applied retrospectively for the analysis. Future studies should include a more balanced set of questions to enable advanced comparisons between cohorts.

## Conclusion

The findings indicate that careful assessment setup choices can and should be used to foster conscious CR choices in students. Digital assessments have additional potential to effectively visualize CR. This large-scale CR visualization may complement the quality screening of MCQ, especially for future high-stakes exams. Further research into large scale CR visualization may help to better understand student performance differences and it may help individualize standardized assessments. CR visualization may also help align MCQ CR with clinical practice and therefore increase its value for workplace training.

## Abbreviations

- WS: Winter semester
- SS: Summer semester
- CR: Clinical reasoning
- FR: Forward reasoning
- BR: Backward reasoning
- IMPP: Institut für medizinische und pharmazeutische Prüfungsfragen (German Institute for State Examinations in Medicine, Pharmacy, Dentistry and Psychotherapy (IMPP))
- MCQ: Multiple choice question
- KFQ: Key feature question
- Wonca: World Organisation of National Colleges of Family Medicine

## Authors’ ORCIDs

- Alexander Oksche: [0000-0003-4592-1770]
- Fabian Dupont: [0000-0003-2247-5640]

## Competing interests

The authors declare that they have no competing interests. 

FD is current Wonca World executive member and director & Wonca World young doctor lead.
